# Modern Bullet Wounds

**Published:** 1903-08-01

**Authors:** 


### MODERN BULLET WOUNDS.

In the course of an address, in which he described some of his experiences as surgeon with the American army in the Philippines, Dr. W. D. Webb[1] stated that his personal observations of the character of wounds made by the modern small bore high velocity bullets was not in accordance with what he had read in the literature of the subject. He found that he was quite unable to tell from the nature of the wound at what range a wounded man had been hit, or to say beforehand what kind of wound would occur at a given range. As a general rule the nature of the tissues struck was of more consequence than the range at which the bullet was fired. Thus wounds of the cranium or of compact bones were accompanied by extensive comminution, while wounds involving the soft tissues were, almost always, perforating in character with very small apertures of entrance and exit. He saw a number of accidental wounds of the hands and feet where the muzzle of the rifle was within a few inches of the injured member, and in these cases a small perforating wound was the usual result unless the metacarpal or metatarsal bones were involved, in which case there was commonly present extensive comminution with forcing of the fragments outwards and a large wound of exit. He also saw a number of wounds made at 10 to 20 yards in native prisoners shot while attempting to escape, and these, again, failed to show the explosive effect described for this range, unless the cranium was involved. Three natives, shot at fourteen-hundred yards range, sustained perforating wounds with small apertures of entrance and exit, two through the chest, and one through the thigh. Wounds of the head were accompanied by extensive comminution, and usually resulted in immediate death. Wounds of the thorax were in practically all cases perforating with small wounds of entrance and exit. An uneventful recovery with few symptoms other than the expectoration of a small amount of blood was the rule, unless a large vessel was involved, in which case death usually occurred on the field. Wounds of the abdomen—

unlike the experiences of the South African War—were highly fatal. Every case that he saw which was not operated on died either immediately or soon after the wound was inflicted from peritonitis. Only two cases recovered, and in both there were perforations of stomach or intestine which were closed by suture.

[1] Post Graduate, July.

